# Computational Study of CO_2_ Capture by Metallic Nanoparticles in Diethylenetriamine Solution

**DOI:** 10.1021/acsomega.6c03032

**Published:** 2026-09-20

**Authors:** Marcus Vinícius F. Rodrigues, Lucas Lima Bezerra, Leonardo Paes da Silva, Norberto de Kássio Vieira Monteiro

**Affiliations:** † Department of Analytical and Physical Chemistry, Federal University of Ceará, 60020-181 Fortaleza, CE, Brazil; ‡ Departament of Organic and Inorganic Chemistry, Federal University of Ceará, 60020-181 Fortaleza, CE, Brazil

## Abstract

The high concentration of CO_2_ present in the atmosphere caused by human action is triggering a series of climate disorders worldwide, such as an increase in global temperatures. Therefore, it is necessary to develop new efficient and sustainable technologies to remove excess CO_2_ gas from the atmosphere. Based on this problem, this work aims to investigate the action of noble metal nanoparticles contained in diethylenetriamine (DETA) on CO_2_ capture through computational simulations. First, platinum nanoparticles (PtNPs) and palladium nanoparticles (PdNPs) are obtained by the CHARMM-GUI server, and DETA, CO_2_, and H_2_O optimizations are based on Density Functional Theory (DFT). Finally, from Molecular Dynamics (MD), the configurations of Interaction Potential Energy (IPE), Radial Distribution Function (RDF), Spatial Distribution Function (SDF) and weak interactions analysis, Non-Covalent Interactions (NCI) and Thermal Fluctuation index (TFI) are used to confirm the best system for CO_2_ capture. The simulations indicate that PtNP–DETA–H_2_O–CO_2_ is the best system, showing the longest stability in the root mean square deviation (RMSD) analysis, the lowest IPE (−16003.61 kJ mol^–1^); the PtNP in this system has a superior *g*(*r*) when compared to the PdNP, and spatial distribution in SDF analysis and the presence of strong interactions and stability between CO_2_ and DETA are demonstrated in aIGM and TFI images, respectively. These results suggest future application of these materials in a practical structure, like industrial plants.

## Graphical abstract

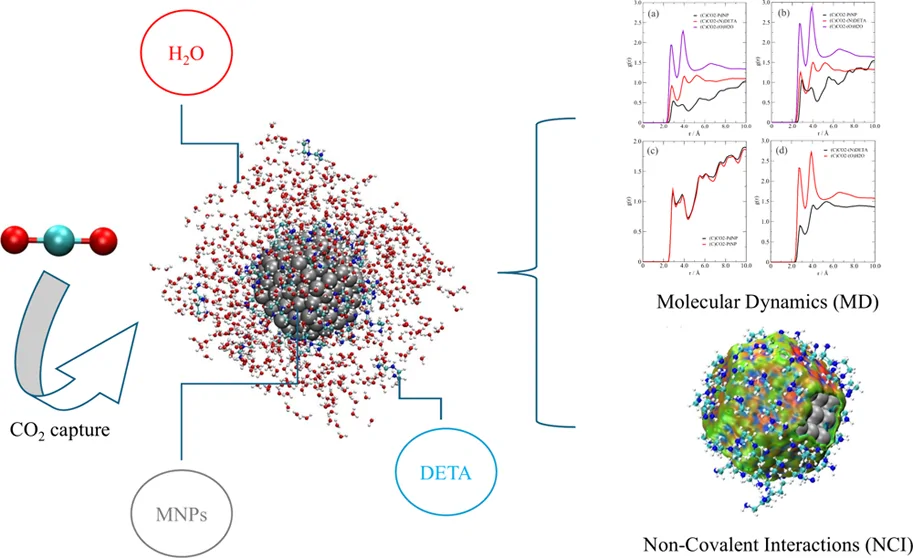

## 1 Introduction

Nowadays, one of the biggest challenges is the gradual increase in the global temperature. The main cause of this thermal imbalance is greenhouse gas emissions, which are intensified by anthropic actions like consumption of fossil fuels and forest deforestation.^1,2^ The greenhouse effect is the heat retention from gases like methane (CH_4_), water vapor (H_2_O), and carbon dioxide (CO_2_).^3^ For this reason, the life of various fauna and flora is possible because this effect allows for a milder temperature in the atmosphere.^4,5^ However, the increase in the concentration of these gases, primarily CO_2_, which is a product of the combustion of fossil fuels such as oil derivatives and mineral coal, generates a deregulation in temperature in the atmosphere. This increase was studied by the Mauna Loa Observatory, which showed that the acceptable limit of CO_2_ in the atmosphere (350 ppm) was exceeded in 2022 (421 ppm).^6,7^

This fuel combustion occurs, for the most part, due to improvements in world energy consumption since the energy matrices used are oil and its derivatives (29.5%), mineral coal (27.2%), and natural gas (23.6%), according to the International Energy Agency (IEA).^8^ Besides, the main fuels used in most common vehicles are oil derivatives such as gasoline, diesel, natural gas, and aviation fuel.^9,10^ Therefore, developing new greenhouse gas capture technologies, especially for CO_2_, is necessary to reduce global warming and alleviate the climate crisis.

In Carbon Capture and Storage (CCS), the process involves four categories: precombustion, chemical looping combustion, oxyfuel combustion, and postcombustion.^11–15^ The latter is the most adaptable and recommended for studies on generating gas sources.^16^ One option for postcombustion CCS is amine-based solvents like diethylenetriamine (DETA).^17–19^ This solvent presents low reactivity compared to reference solvents like monoethanolamine (MEA), which has high volatility and causes damage to industrial equipment.^11,20,21^ Another aspect to consider is the CO_2_ absorption kinetics and the CO_2_ absorption capacity of DETA, which is superior to MEA due to having more amine groups.^22–24^ Wu et al.^25^ studied DETA modified with hydroxyalkyls for CO_2_ absorption. Their computational analysis used the DFT method to determine the Gibbs free energy of initial, transitional, and final states of the reactions using the software Gaussian 16 with the B3LYP/6-311++(2d,p) configuration. The results indicated that 2-hydroxypropyl-DETA presented the best results for CO_2_ capture with a lower free energy barrier and a favorable electrostatic potential (ESP).

Another material with good absorption capacity for CCS is metallic nanoparticles (MNPs), particularly noble metals.^26–30^ These metals have higher catalytic properties in bimetallic alloys or modified surfaces applied in Hydrogen Evolution Reaction (HER) and Oxygen Evolution Reaction (OER) in fuel cells or electrocatalysts.^31–33^ Besides, these noble metals have applications in other reactions, such as gold used as a catalyst in hydrogen production in the water–gas shift process,^34^ beyond platinum, palladium, and rhodium present in automotive exhaust catalysts.^33^ Li et al.^35^ studied the performance of Na-promoted (Pt–Na) Pt nanoparticles supported in Al_2_O_3_ for CO_2_ capture and selective hydrogenation. The analysis of catalyst tests confirmed the best adsorption for CO_2_ and selectivity for CO formation at high temperatures (623.15 K) when compared to Mg addition.

CO_2_ capture with MNPs requires solvents for NP formation, such as ionic liquids (IL)^36,37^ and deep eutectic solvents (DES).^38–41^ These solvents stabilize the thermodynamic instability of MNPs, avoiding aggregation and preserving their catalytic behavior.^42,43^ Although there are no reports indicating that DETA can serve as a stabilizing agent, the presence of amine groups in both IL and DES suggests its possible use as a stabilizing agent.

Torkzadeh and Moosavi^44^ studied the interaction between dicationic ionic liquids (DILs) and CO_2_. The DILs [Bis­(mim)­C_5_]­[NTf_2_]_2_, [Bis­(mim)­C_5_-(C_4_)_2_]­[NTf_2_]_2_ and [N_111_-C_5_-mim]­[NTf_2_]_2_ were analyzed to verify the effect of cation symmetry and chain length, and the analysis showed that ([Bis­(mim)­C_5_-(C_4_)_2_]­[NTf_2_]_2_) interacted better with CO_2_. Shaikh et al.^45^ analyzed choline-based IL amino acids (glycine, alanine, serine, and proline) and other anions (−COOH, −NH_2_, and −OH) with DMSO for CO_2_ capture with MD and DFT analysis. Ghaed-Sharaf and Omidvar^46^ developed a study with green IL cholinium geranate and DES cholinium geranate, through MD and DFT, which revealed higher interaction for the cholinium geranate IL than the cholinium geranate DES. Li et al.^47^ analyzed metal-based ionic liquids (MBILs: [Bmim]­[Cl] and [Bmim]­[FeCl_4_], [Bmim]_2_[ZnCl_4_], [Bmim]_2_[CuCl_4_], and [Bmim]­[CrCl_4_]) to determine their mechanism of capture of CO_2_ and the effect of metal in absorbing CO_2_ through MD. It was observed that the highest interactions were between [Bmim]­[Cl] and [Bmim]_2_[ZnCl_4_] and CO_2_. Yang et al.^48^ used DFT to study the mechanism for CO_2_ capture and conversion into methane for photoreduction with Pd MNPs supported on a TiO_2_ surface, showing that the presence of MNPs improved the reaction performance. Liu et al.^49^ reported a study involving the use of an amino acid (l-Met) to form a CO_2_ hydrate from CO_2_ liquid, a clathrate for CO_2_ capture in offshore structures. The computational simulations indicated that l-Met showed a reduction in energy barrier when compared with water and a preferential approximation of CO_2_ on the hydrophobic amino acid part.

These studies showed a direct relationship between amine-based solvents and MNPs for CO_2_ capture; however, works involving MNPs with amine solvents for CO_2_ capture through a computational approach still remain poorly explored. Therefore, this work relates the use of Pd and Pt MNPs (PdNPs and PtNPs, respectively) in a DETA solution for CO_2_ capture through computational simulations. Using MD, the goal was to understand the molecular interactions among CO_2_, DETA, MNPs, and H_2_O and identify which interactions are the strongest with CO_2_. The systems are divided into MNP–DETA–H_2_O–CO_2_, MNP–CO_2_, and DETA–H_2_O–CO_2_ and compared to determine the best CO_2_ capture system.

## 2 Computational Details

### 2.1 Molecular Structures

The structures of DETA, CO_2_, and H_2_O were optimized using Density Functional Theory (DFT) calculations, which were applied to determine the lowest energy tridimensional structure using B3LYP^50–52^ as a hybrid functional with the basis set 6-311+G­(d,p),^53^ implemented in the Gaussian 09 ^54^ software. The PdNPs and PtNPs were based on the Atilhan and Aparicio^55^ study. The MNPs were spherical, and the number of atoms was 201 for each. The diameters were 17.8 Å for PdNPs and 17.9 Å for PtNPs. First, the MNPs were built in the CHARMM-GUI server^56^ in the Nanomaterial Modeler module,^57^ and the topology files were converted for the OPLS-AA force field^58^ by the TopolGen 1.1_dev script written by Lemkul.^59^ Figure 1 shows the structures of the components used in this work.

**Figure 1:**
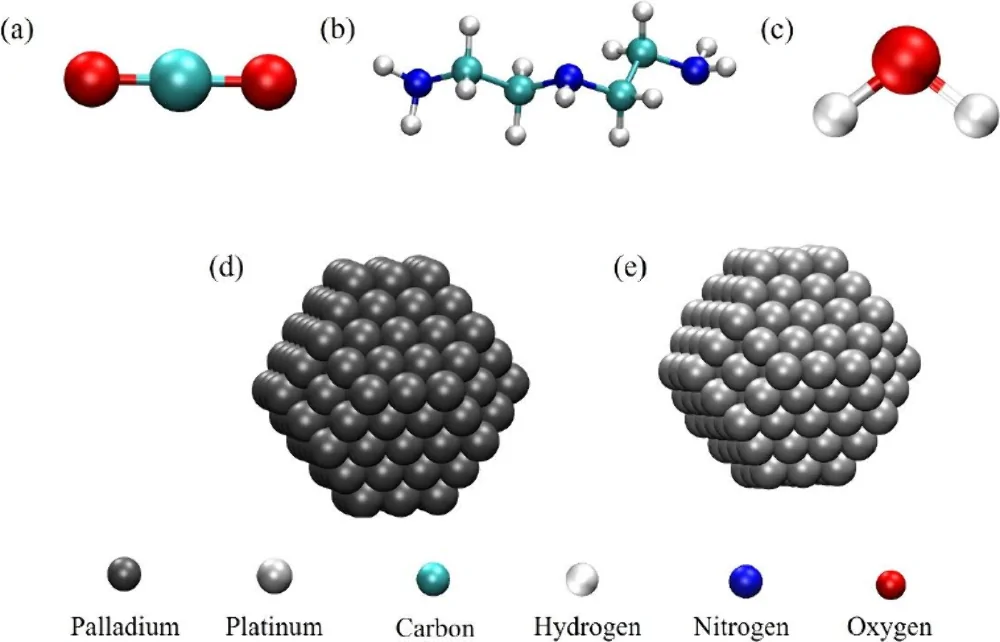
Illustration of (a) CO_2_, (b) DETA, (c) H_2_O, (d) PdNP, and (e) PtNP.

### 2.2 Molecular Dynamics

All simulations were performed using the GROMACS software 2021.2 ^60^ with the OPLS-AA force field,^58^ used in previous work involving gas capture.^61,62^ The CO_2_ parameters were obtained from Zhu et al.,^63^ and water molecules were described by the TIP3P method.^64^ The 36 DETA, 804 H_2_O, and 285 CO_2_ numbers were based on the experimental study of Zhang et al.,^65^ and the simulations were performed in a cubic box with initial dimensions 12 × 12 × 12 nm^3^. The systems were divided into three groups: MNP–DETA–H_2_O–CO_2_ (PtNP–DETA–H_2_O–CO_2_ and PdNP–DETA–H_2_O–CO_2_), MNP–CO_2_ (PtNP–CO_2_ and PdNP–CO_2_), and DETA–H_2_O–CO_2_.

The system’s geometry was optimized using the steepest descent^66^ and the gradient conjugate algorithms,^67^ with an energy tolerance of 10 kJ mol^–1^, 10^5^ steps, and 10^–4^ nm step size. Then, the equilibration was developed by using NVT and NPT ensembles for 400 ps using the LINCS algorithm constraint,^68^ the Particle Mesh Ewald (PME) for long-range interactions,^69^ and 1.0 nm as the cutoff radius for Coulombic and van der Waals short-range interactions under the periodic boundary conditions (PBC). The NVT ensemble was the first step with the V-rescale thermostat^70^ at 303.15 K for all systems, and the second step, the NPT ensemble, utilized the C-rescale barostat^71^ with a pressure of 1.029 bar. Finally, the production stage was realized with the Leap-Frog algorithm^72^ in 100 ns with the parameters of the equilibration stage. Additionally, Visual Molecular Dynamics (VMD) 1.9.3 ^73^ was used to visualize MD results; each analysis was performed in triplicate.

The Interaction Potential Energy (IPE)^74,75^ was determined from the sum of the short-range Lennard-Jones and Coulomb energies according to eq 1. These energies quantify the affinity between CO_2_ and MNPs, DETA, and H_2_O molecules. The *N*_*i*_ and *N*_*j*_ values are the total number of atoms selected for analysis. *V*_vdW_ and *V*_elec_ are related to van der Waals and electrostatic contributions, respectively.

1 $${\mathrm{IPE}}_{ij}=\overset{N_{i}}{\underset{i}{\sum }}\overset{N_{j}}{\underset{j\neq i}{\sum }}V_{\mathrm{vdW}}\left(r_{ij}\right)+V_{\mathrm{elec}}\left(r_{ij}\right)$$

### 2.3 Weak Interactions Analysis

To identify the weak interactions^76^ between MNPs and DETA, H_2_O, and CO_2_, the average independent gradient model (aIGM) was used through Multiwfn 3.8 ^77^ software. This analysis used noncovalent interactions (NCIs), which are illustrated as isosurfaces in selected regions to define the weak intermolecular forces. The MNPs were selected to define isosurfaces and classify interactions with DETA, H_2_O, and CO_2_ as follows: strong interactions are indicated by blue color, van der Waals interactions by green color, and repulsive interactions by red color. Thermal fluctuation interaction (TFI) analysis identified the stability of interactions with highly stable ones represented in blue, moderately stable ones represented in green, and weakly stable ones indicated by red color (Figures S1–S3). Finally, the resulting images were generated by the VMD software.

## 3 Results and Discussion

### 3.1 Root Mean Square Deviation (RMSD)

RMSD analysis was applied to systems to determine their equilibrium in a range of 0 to 100 ns, represented in Figure 2. This equilibrium indicates the stability of the system’s components, and the reference selected in this analysis was the entire system. In the PdNP–DETA–H_2_O–CO_2_ system (Figure 2a), equilibrium was reported at approximately 40 ns, while in the PtNP–DETA–H_2_O–CO_2_ system (Figure 2b) and PtNP–CO_2_ system (Figure 2d) it was reached at 30 ns, in the PdNP–CO_2_ system (Figure 2c) it was reached at 80 ns, and in the DETA–H_2_O–CO_2_ system (Figure 2e) it was reached in 50 ns. The PtNP–DETA–H_2_O–CO_2_ system, Figure 2b, reached equilibrium more quickly than other systems, which indicated that it was the most stable. Furthermore, the insertion of MNPs contributed to reducing RMSD values in a DETA–H_2_O–CO_2_ system, indicating good system stability.

**Figure 2:**
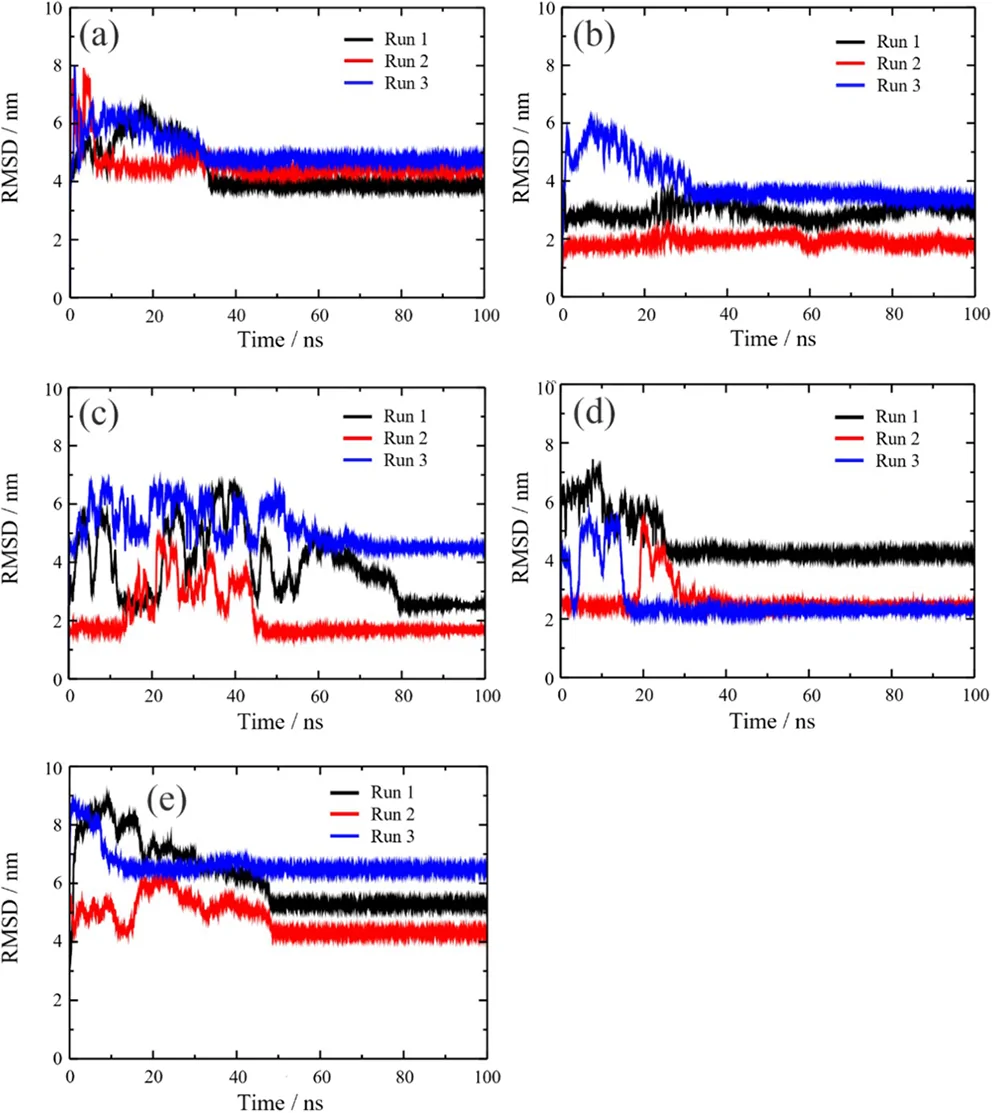
RMSD of PdNP–DETA–H_2_O–CO_2_ (a), PtNP–DETA–H_2_O–CO_2_ (b), PdNP–CO_2_ (c), PtNP–CO_2_ (d) and DETA–H_2_O–CO_2_ (e). The black, red, and blue lines represent simulated replicates.

### 3.2 Average IPE Values

Table 1 presents the average IPE values for the five systems to determine the best interaction with CO_2_ and, consequently, the best system for CO_2_ capture, starting from the equilibrium time indicated by the RMSD results. In MNP–CO_2_ systems, IPE average values were found to be more negative for PtNPs (−4560.20 kJ mol^–1^) when compared to PdNPs (−3915.87 kJ mol^–1^). PtNP–DETA–H_2_O–CO_2_ demonstrated the highest interactions with CO_2_ (−16003.61 kJ mol^–1^) compared with PdNP–DETA–H_2_O–CO_2_ (−15867.81 kJ mol^–1^). Besides, a synergistic effect of MNPs when combined with DETA–H_2_O was observed, as shown by energy increase compared to the DETA–H_2_O–CO_2_ interaction (−15194.25 kJ mol^–1^). These results suggest that the PtNP–DETA–H_2_O–CO_2_ system with lower IPE was the best system for CO_2_ capture. Even though the IPE value of PtNPs is lower than those of DETA and H_2_O, the relative contributions of each component account for this disparity, and the MNPs can alter the molecular distribution in the simulation box, thereby permitting synergistic effects and modifying the capture behavior.

**Table 1:** Average IPE Values (kJ mol^–1^) of Interactions between CO_2_ and PdNP, PtNP, DETA, and H_2_O for Each System

	Interactions				
Systems	NP–CO_2_	DETA–CO_2_	H_2_O–CO_2_	NP–DETA–H_2_O–CO_2_	DETA–H_2_O–CO_2_
PdNP–DETA–H_2_O–CO_2_	–728.05 (±26.52)	–1375.85 (±12.30)	–13769.87 (±28.06)	–15867.81 (±21.16)	
PtNP–DETA–H_2_O–CO_2_	–981.61 (±34.06)	–1392.19 (±3.16)	–13614.20 (±26.50)	–16003.61 (±49.26)	
PdNP–CO_2_	–3915.87 (±166.88)				
PtNP–CO_2_	–4560.20 (±93.91)				
DETA–H_2_O–CO_2_		–1503.05 (±3.46)	–13691.20 (±3.24)		–15194.25 (±1.36)

### 3.3 Radial Distribution Function (RDF)

In RDF analysis, the carbon of CO_2_ was selected for the RDF reference to determine the density of probability around CO_2_ carbon and determine the most probable component according to refs 78 and 79 . In Figure 3a, the PdNP–DETA–H_2_O–CO_2_ was found to have higher *g*(*r*) for the oxygen of H_2_O (CO_2_–H_2_O) followed by DETA nitrogen (CO_2_–(N)­DETA) and the PdNP (CO_2_–PdNP). The highest *g*(*r*) of CO_2_ interacting with water presents two solvation layers in 3.0 Å and 4.0 Å, Figure 3a,b,d), due to the higher amount in the system and even their high solubility. In DETA, the three nitrogens of the molecule presented a strong interaction with CO_2_, represented by three solvation layers (Figure 3a,b,d). In Figure 3b, the PtNP–DETA–H_2_O–CO_2_ system, the behavior of interactions was similar to that presented in Figure 3a, with the densities of the components higher when compared to those of the PdNP–DETA–H_2_O–CO_2_ system. In Figure 3c, the CO_2_–PdNP and CO_2_–PtNP systems exhibited similar profiles with a solvation layer in 3.0 Å and 4.0 Å and a rise in *g*(*r*). Figure 3d represents the DETA–H_2_O–CO_2_ system, indicating two solvation layers of CO_2_–H_2_O and three of CO_2_–DETA. Compared to Figure 3a,b, the system profiles of DETA and H_2_O have the same behavior.

**Figure 3:**
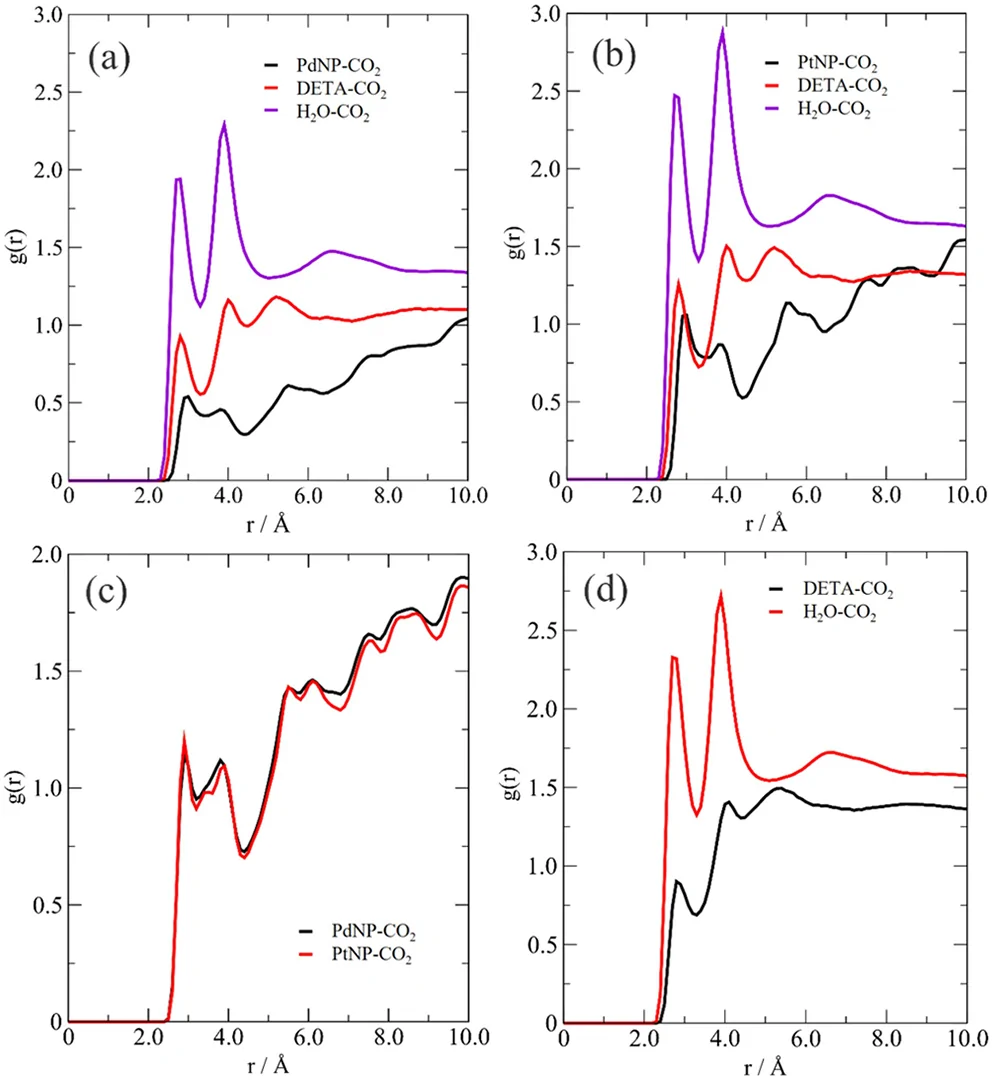
RDF of PdNP–DETA–H_2_O–CO_2_ (a), PtNP–DETA–H_2_O–CO_2_ (b), PdNP–CO_2_ and PtNP–CO_2_ (c), and DETA–H_2_O–CO_2_ (d) systems.

### 3.4 Spatial Density Function (SDF)

Figure 4 illustrates the spatial probability distribution of a component analyzed using SDF analysis.^80,81^ As with the RDF, one CO_2_ molecule was selected in each system to verify the highest SDF around CO_2_ and to assess similarity with the RDF results, thereby revealing the highest spatial probability of a component and, consequently, the highest interaction. The PdNP–DETA–H_2_O–CO_2_ system (Figure 4a) had major density of water (purple color) around the CO_2_, followed by DETA (green color) and PdNP (orange color), indicating similarity to the RDF analysis (Figure 3a). Similar behavior was observed in the PtNP–DETA–H_2_O–CO_2_ system (Figure 4b), where the water spatial density was predominant followed by the DETA molecule and PtNP (blue), which showed a higher density than that of PdNP, suggesting a stronger interaction. The PdNP–CO_2_ system (Figure 4c) showed similar interactions with CO_2_, while the PtNP–CO_2_ system (Figure 4d), with a lower spatial density, showed similar interactions with CO_2_, and the DETA–H_2_O–CO_2_ system (Figure 4e) presented a higher spatial density for water and a lower one for DETA, as shown in Figures 4a,b. All of these results are confirmed by RDF and IPE analysis, since the most probable interacting molecule is water, followed by DETA and metallic MNPs.

**Figure 4:**
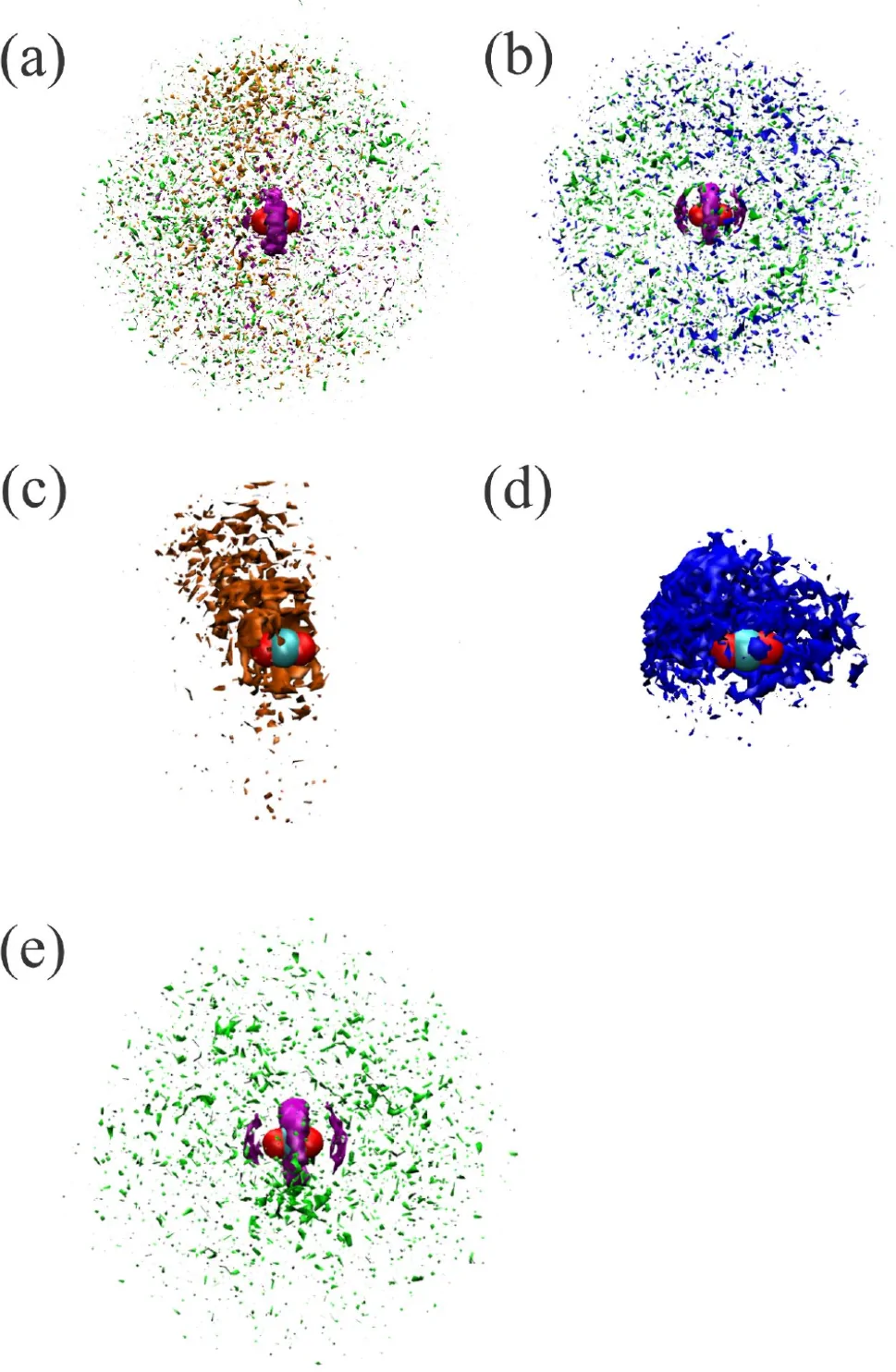
SDFs of PdNP–DETA–H_2_O–CO_2_ (a), PtNP–DETA–H_2_O–CO_2_ (b), PdNP–CO_2_ (c), PtNP–CO_2_ (d), and DETA–H_2_O–CO_2_ (e) systems.

### 3.5 Averaged Independent Gradient Model (aIGM)

The aIGM analysis of the PdNP and other components of the PdNP–DETA–H_2_O–CO_2_ system was performed during all the simulations, and the isosurfaces are shown in Figure 5. This method was utilized to reveal the type of predominant intermolecular interaction throughout the MD simulation with the MNPs. The blue color represents strong interactions such as hydrogen bonds; the green color represents van der Waals interactions, and the red color represents repulsive interactions such as steric effects.

**Figure 5:**
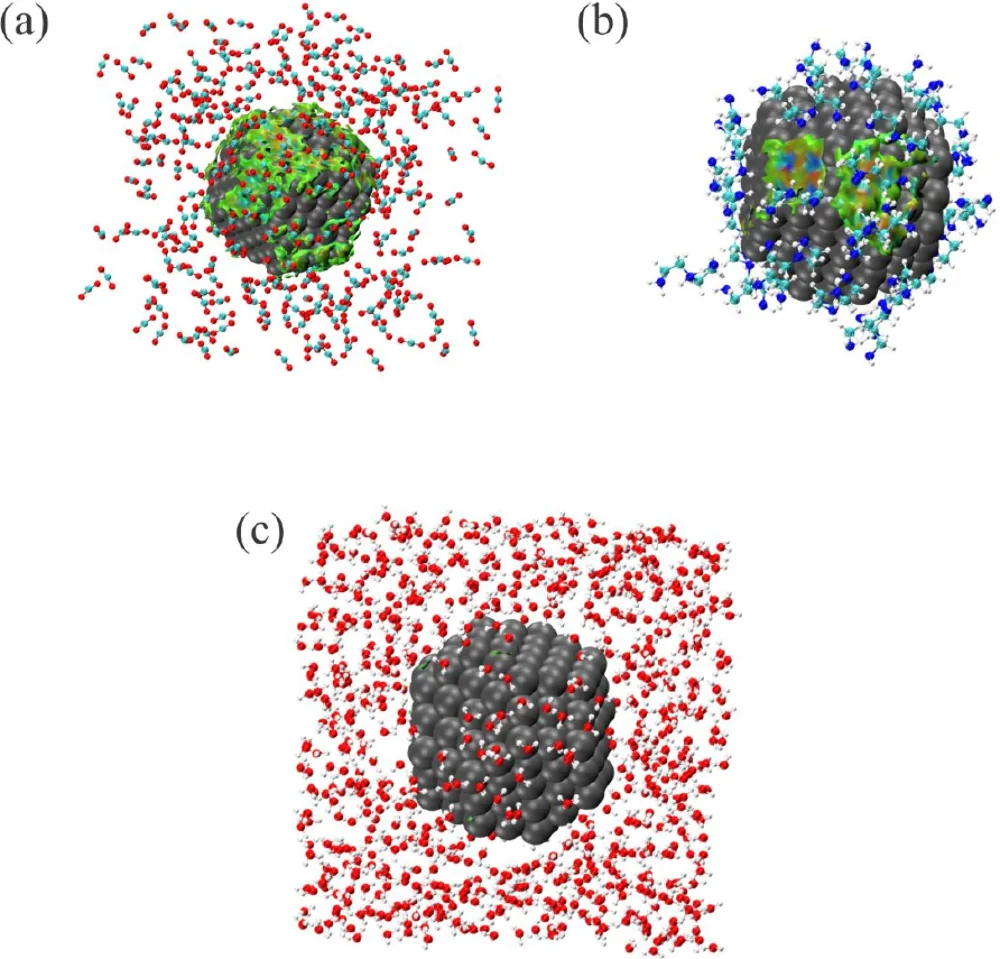
aIGM isosurfaces of the PdNP–DETA–H_2_O–CO_2_ system between the NP and CO_2_ (a), DETA (b), and H_2_O (c).

The isosurfaces show the dominance of van der Waals interactions in CO_2_ and DETA, prevailing over interactions with CO_2_ alone, followed by DETA and H_2_O, indicating a low interaction. The CO_2_ isosurface shows good interaction with the PdNP, presenting effective capture behavior but does not indicate interactions with the nanoparticle and DETA, compromising its activity. The DETA molecule can act as a stabilizing agent in the nanoparticle, preventing aggregation with other MNPs and thereby improving its adsorbing activity. Figure 6 illustrates the interactions of the PtNP isosurface in the PtNP–DETA–H_2_O–CO_2_ system with DETA, H_2_O and CO_2_.

**Figure 6:**
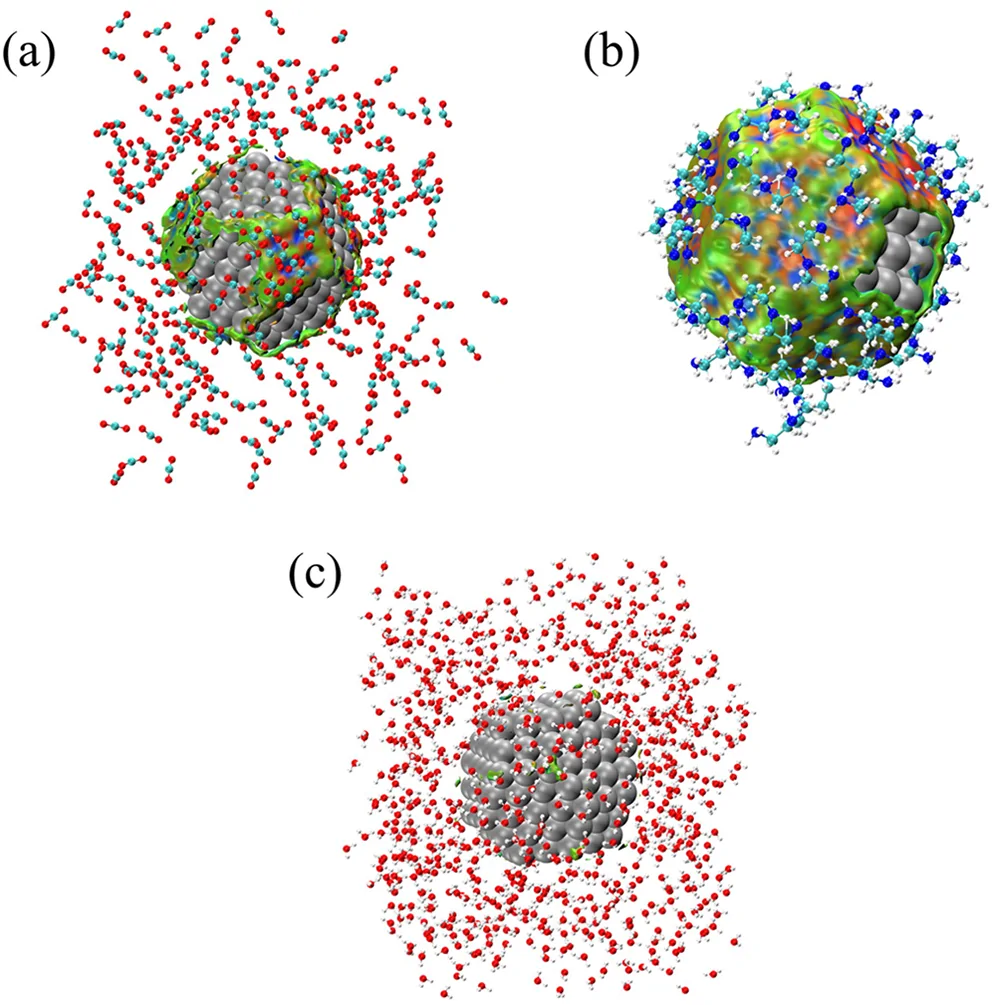
aIGM isosurface of PtNP–DETA–H_2_O–CO_2_ system between the NP and CO_2_ (a), DETA (b), and H_2_O (c).

In the PtNP system, the DETA isosurface (Figure 6b) showed a homogeneous distribution of DETA with van der Waals interactions being dominant but also some points in red and blue. The CO_2_ isosurface (Figure 6a) proved to be smaller than that with the PdNP (Figure 5a), therefore revealing blue colors, indicating the presence of strong interactions, and the H_2_O isosurfaces (Figure 6c) are irrelevant, as they were in the PdNP system (Figure 5c).

The effective DETA interaction with the PtNP explains the high IPE value in Table 1, where DETA and PtNP show a synergistic effect for CO_2_ capture. Furthermore, this suggests that DETA could serve as a possible stabilizing agent for PtNP synthesis. In Figure 7, the aIGM of the PdNP–CO_2_ and PtNP–CO_2_ systems is presented.

**Figure 7:**
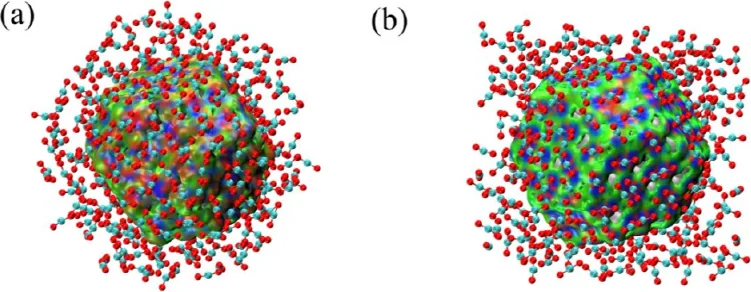
aIGM isosurface of PdNP (a) and PtNP (b) with CO_2_.

The PtNP–CO_2_ isosurface (Figure 7b) appears predominantly green and blue, dispersed throughout the nanoparticle volume, indicating van der Waals and strong interactions. Although a similar effect was observed for the PdNP (Figure 7a), the isosurface colors differed, with the PtNP exhibiting colors more intense than the PdNP, which showed predominantly red hues, indicating repulsive interactions. In Figure S3, the TFI analysis showed the PtNP with a presence of blue color in its isosurface, indicating strong stability with CO_2_, which can explain the good interaction with CO_2_.

## 4 Conclusions

The simulations revealed that the PdNP, PtNP, DETA, and H_2_O interact favorably with CO_2_, with the PtNP–DETA–H_2_O–CO_2_ system exhibiting the strongest interaction. For the PtNP–DETA–H_2_O–CO_2_ system, the RMSD graph indicates the most stable system, IPE values exhibit lower energy, RDF analysis shows higher *g*(*r*), and SDF analysis illustrates the highest spatial density. Furthermore, the aIGM images showed stronger interactions between PtNPs and CO_2_ and DETA molecules around the MNPs, indicating a possible stabilizing agent for CO_2_ capture, and TFI analysis confirmed the highest stability interaction of PtNPs with CO_2_, and the DETA molecules in the PtNP–DETA–H_2_O–CO_2_ system were more distributed than those in the PdNP–DETA–H_2_O–CO_2_, showing a stabilization between PtNPs and DETA, which can contribute to their synergetic effect.

## Supplementary Material



## Notes

TFI isosurfaces of MNPs for the PdNP–DETA–H_2_O–CO_2_, PtNP–DETA–H_2_O–CO_2_, PdNP–CO_2_, and PtNP–CO_2_ and the IPE replicates of each component with CO_2_ (PDF)
