# Supplementary material for: Computational Study of CO2 Capture by Metallic Nanoparticles in Diethylenetriamine Solution
Source: ACS Omega. 2026 Sep 20;11(39):58765–72. doi: 10.1021/acsomega.6c03032 (PMC13647427; doi:10.1021/acsomega.6c03032)
Supplement: Supplementary file 1 [file ao6c03032_si_001.pdf]

COMPUTATIONAL STUDY OF CO_2 CAPTURE BY METALLIC NANOPARTICLES IN DIETHYLENETRIAMINE SOLUTION

Marcus Vinícius F. Rodrigues[a], Lucas Lima Bezerra[b], Leonardo Paes da Silva[a], Norberto de Kássio Vieira Monteiro[a*]

[a] *Department of Analytical and Physical Chemistry, Federal University of Ceará, 60020-181, Fortaleza-CE, Brazil*

[b] *Department of Organic and Inorganic Chemistry, Federal University of Ceará, 60020-181, Fortaleza-CE, Brazil*

***Corresponding author.** Email Address: norbertokv@ufc.br

Phone: +55 85 3366 9980

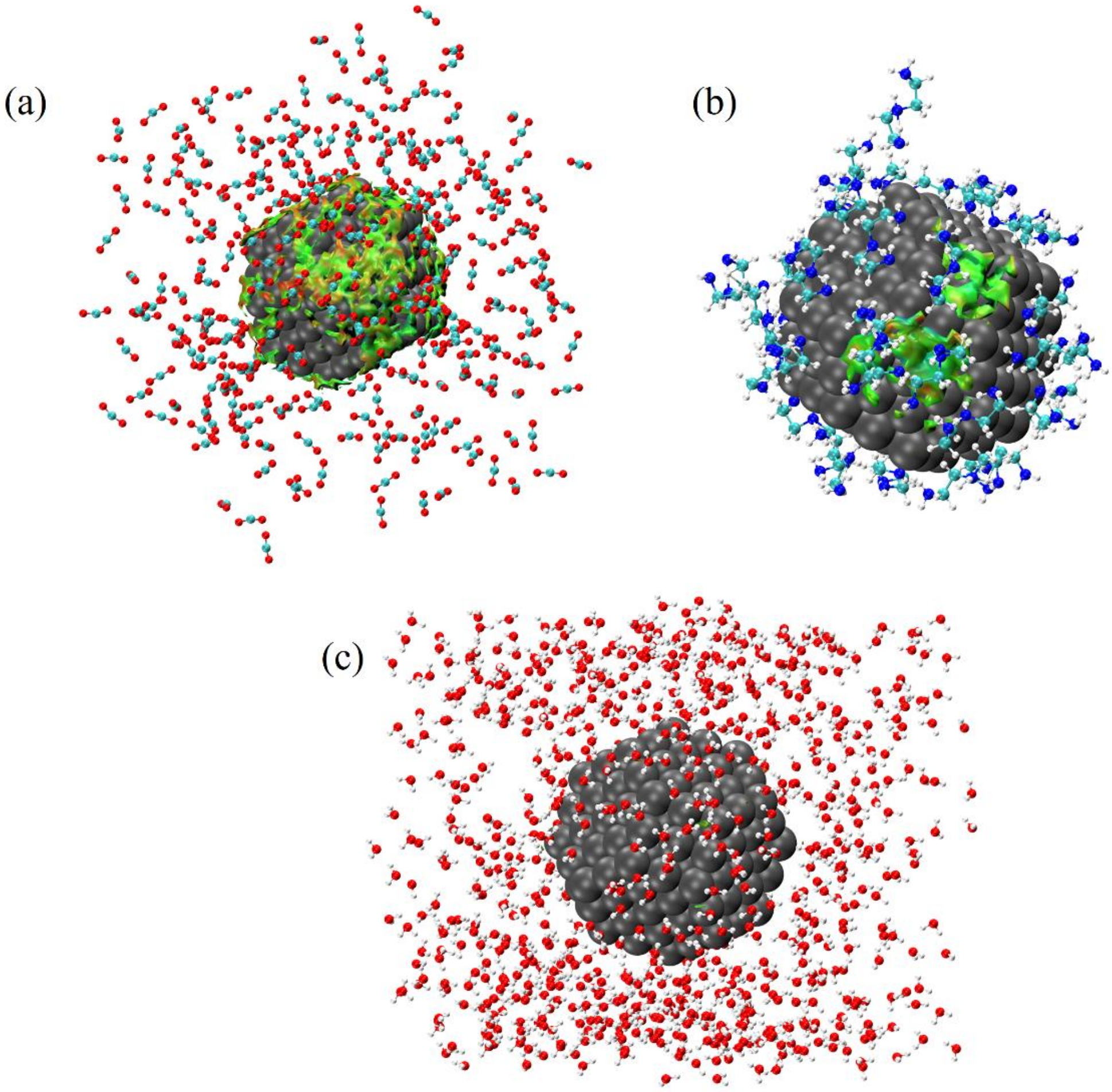

Figure S1: TFI isosurfaces of PdNP-DETA-H_2O-CO_2 system between CO_2 (a), DETA (b) and H_2O (c)

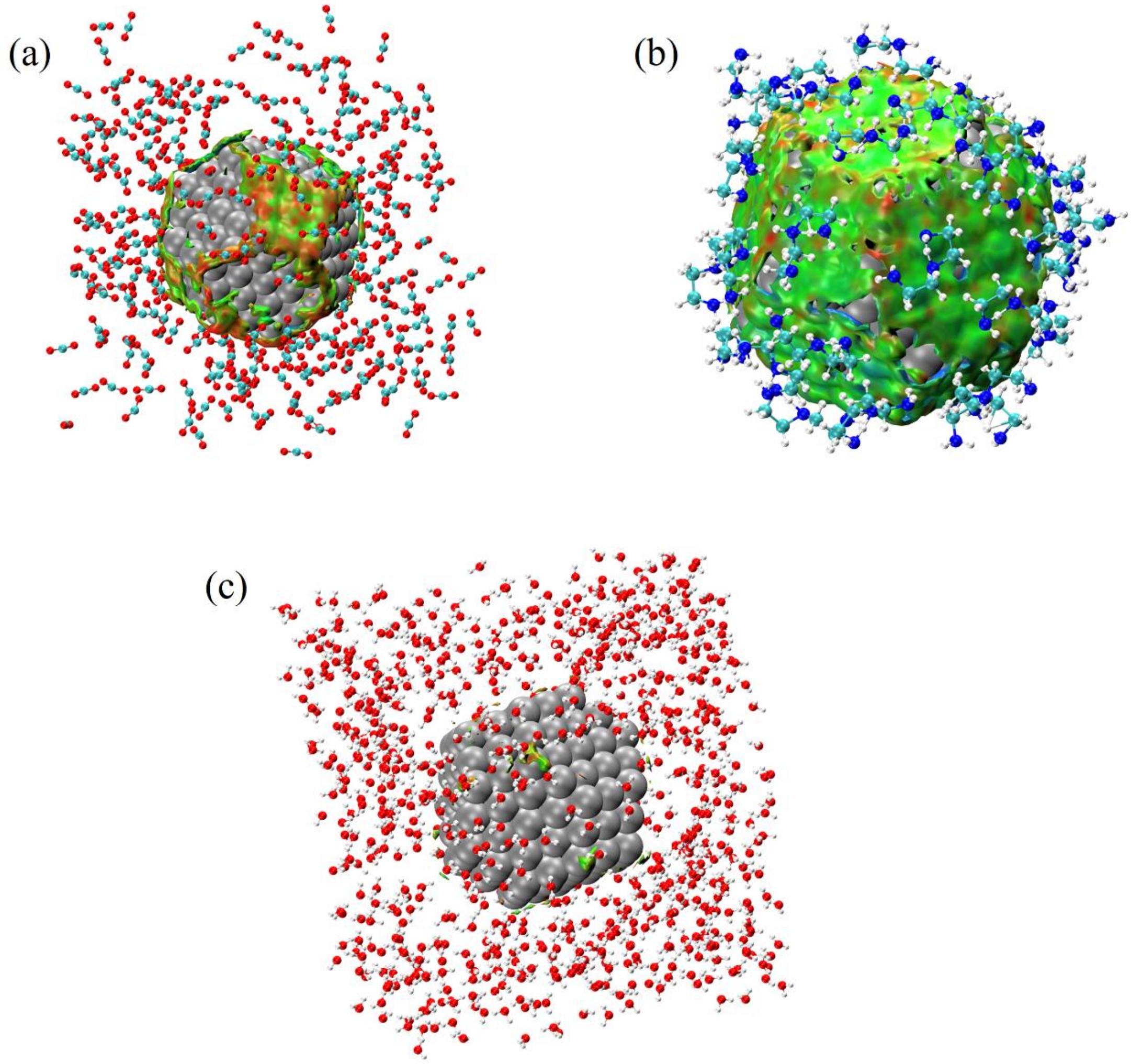

Figure S2: TFI isosurfaces of PtNP-DETA-H_2O-CO_2 system between CO_2 (a), DETA (b) and H_2O (c)

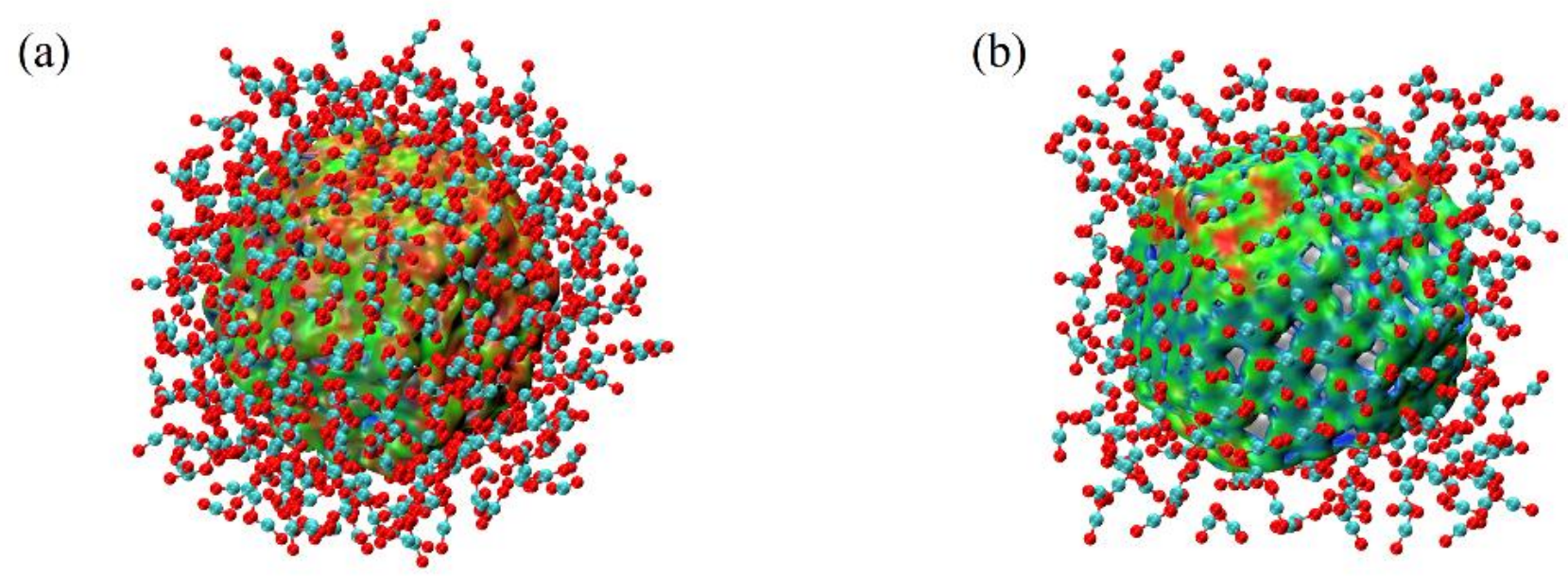

Figure S3: TFI isosurfaces of PdNP (a) and PtNP (b) between CO_2.

Table S1: IPE values (kJ mol^{-1}) of the Replicate #1 for MNPs, DETA and H_2O with CO_2.

Systems/Interactions	NP-CO_2	DETA-CO_2	H_2O-CO_2	NP-DETA-H_2O-CO_2	DETA-H_2O-CO_2
PdNP-DETA-H_2O-CO_2	−720.655	−1391.850	−13779.500	−15892.005	
PtNP-DETA-H_2O-CO_2	−1010.160	−1388.810	−13559.300	−15958.27	
PdNP-CO_2	−3980.710				
PtNP-CO_2	−4454.76				
DETA-H_2O-CO_2		−1500.180	−13694.700		−15194.880

Table S2: IPE values (kJ mol^{-1}) of the Replicate #2 for MNPs, DETA and H_2O with CO_2.

Systems/Interactions	NP-CO_2	DETA-CO_2	H_2O-CO_2	NP-DETA-H_2O-CO_2	DETA-H_2O-CO_2
PdNP-DETA-H_2O-CO_2	−757.483	−1371.520	−13729.700	−15858.703	
PtNP-DETA-H_2O-CO_2	−990.761	−1392.68	−13613.1	−15996.54	
PdNP-CO_2	−3798.950				
PtNP-CO_2	−4591.000				
DETA-H_2O-CO_2		−1506.890	−13688.300		−15195.190

Table S3: IPE values (kJ mol^{-1}) of the Replicate #3 for MNPs, DETA and H_2O with CO_2.

Systems/Interactions	NP-CO_2	DETA-CO_2	H_2O-CO_2	NP-DETA-H_2O-CO_2	DETA-H_2O-CO_2
PdNP-DETA-H_2O-CO_2	−706.000	−1369.730	−13777.000	−15852.730	
PtNP-DETA-H_2O-CO_2	−943.901	−1395.07	−13670.2	−16056.031	
PdNP-CO_2	−3967.930				
PtNP-CO_2	−4634.850				
DETA-H_2O-CO_2		−1502.090	−13690.600		−15192.690
